# A Novel Synthetic TLR-4 Agonist Adjuvant Increases the Protective Response to a Clinical-Stage West Nile Virus Vaccine Antigen in Multiple Formulations

**DOI:** 10.1371/journal.pone.0149610

**Published:** 2016-02-22

**Authors:** Neal Van Hoeven, Sharvari Waghmare Joshi, Ghislain Ismael Nana, Angela Bosco-Lauth, Christopher Fox, Richard A. Bowen, David E. Clements, Timothy Martyak, D. Elliot Parks, Susan Baldwin, Steven G. Reed, Rhea N. Coler

**Affiliations:** 1 Infectious Disease Research Institute, 1616 Eastlake Ave E., Seattle, WA 98103, United States of America; 2 Colorado State University Department of Biomedical Sciences, Foothills Campus, Fort Collins, CO 80523, United States of America; 3 Hawaii Biotech Inc. 99-193 Aiea Heights Drive, Aiea, Hawaii 96701, United States of America; Thomas Jefferson University, UNITED STATES

## Abstract

West Nile virus (WNV) is a mosquito-transmitted member of the *Flaviviridae* family that has emerged in recent years to become a serious public health threat. Given the sporadic nature of WNV epidemics both temporally and geographically, there is an urgent need for a vaccine that can rapidly provide effective immunity. Protection from WNV infection is correlated with antibodies to the viral envelope (E) protein, which encodes receptor binding and fusion functions. Despite many promising E-protein vaccine candidates, there are currently none licensed for use in humans. This study investigates the ability to improve the immunogenicity and protective capacity of a promising clinical-stage WNV recombinant E-protein vaccine (WN-80E) by combining it with a novel synthetic TLR-4 agonist adjuvant. Using the murine model of WNV disease, we find that inclusion of a TLR-4 agonist in either a stable oil-in-water emulsion (SE) or aluminum hydroxide (Alum) formulation provides both dose and dosage sparing functions, whereby protection can be induced after a single immunization containing only 100 ng of WN-80E. Additionally, we find that inclusion of adjuvant with a single immunization reduced viral titers in sera to levels undetectable by viral plaque assay. The enhanced protection provided by adjuvanted immunization correlated with induction of a Th1 T-cell response and the resultant shaping of the IgG response. These findings suggest that inclusion of a next generation adjuvant may greatly enhance the protective capacity of WNV recombinant subunit vaccines, and establish a baseline for future development.

## Introduction

West Nile virus (WNV) is a mosquito-borne member of the family *Flaviviridae* that has emerged in recent years to become a serious public health threat. The virus was initially identified in the West Nile district of Uganda in 1937, and has since spread worldwide. West Nile Virus was first identified in North America in the United States in 1999, and has since spread into Canada [1], Mexico [2], as well as central and South America [3]. Following introduction into North America, the number of WNV cases increased steadily as the virus spread geographically; in 2003, almost 10,000 cases were reported in the US, resulting in 264 deaths [4]. Cumulatively between 1999 and 2010 there have been over 780,000 symptomatic cases of WNV in the US. Of these, 16,000 have resulted in neurologic disease, and over 1500 have been fatal [5]. During the 2012 reporting season, the United States reported the second highest number of WNV infections since the outbreak began, with 5674 total cases reported, compared to only 712 cases in 2011 [6]. Serious complications from WNV infection, which result from spread of the virus into the central nervous system (CNS), include meningitis, paralysis, and eventually death (Reviewed in [7, 8]). Infection of the kidneys has also been reported, although the significance of this and contribution to virus induced morbidity remains unclear [9]. The continued geographic spread and consistent seasonal outbreaks of WNV highlight the need for development of effective vaccines.

WNV (family *Flaviviridae*, genus *Flavivirus*) is an enveloped positive-strand RNA virus. The viral genome is translated as a single polypeptide that is co- and post translationally processed to yield the 3 structural and 7 non-structural proteins [10]. The 3 virus structural proteins are the capsid (C) protein and the pre-membrane protein (prM) which is cleaved during virus maturation to yield the membrane (M) protein and envelope (E) protein. The E protein contains the receptor binding and fusion functions of the virus, and an X-Ray crystal structure for the WNV-E protein, as well as many other members of the genus, have been determined [11–14]. Like all *Flavivirus* E proteins, the WNV E-protein can be divided into three distinct structural domains; DI, DII, and DIII. Antibodies to domains DII and DIII have been shown to neutralize the virus, and correlate with resolution of infection in preclinical models [15]. For this reason, the E-protein has been extensively evaluated as a vaccine candidate in both preclinical animal models and in the clinic (Reviewed in [16, 17]). WNV E protein antigen has been delivered as part of an inactivated virus [18–22], as a recombinant protein [23–33], as a DNA vaccine [34–41], as an RNA vaccine [42], and using various replicating and non-replicating viral vectors [43–54]. Live-attenuated vaccines for WNV have also been developed [55–61]. Of the potential vaccine candidates, the live attenuated vaccines have shown promise in the clinic, inducing high levels of virus neutralizing antibodies [62–64]. A recombinant E subunit vaccine, WN-80E, has also been advanced into the clinic, but was found to induce low level neutralizing antibodies when adsorbed to Alum [65]. Given the safety advantages of sub-unit vaccines relative to live attenuated agents, additional development of a WN-80E based vaccine would provide an attractive vaccine candidate.

Vaccine adjuvants are critical for the effective development of protective responses with many antigens. Toll-like receptor (TLR) agonist adjuvants are particularly promising, as they engage the innate immune system to stimulate a more robust and durable adaptive immune response [66]. Ligands for TLR 7/8 (Imiquimod, Resiquimod) [67], TLR 9 (CpG) [68, 69], TLR 5 (Flagellin) [70], and TLR 4 [66, 71, 72] have been evaluated pre-clinically as components of vaccine adjuvants. TLR 9 and TLR 5 have been specifically evaluated in combination with WNV E protein or domain III antigens, and have shown promise in enhancing immunogenicity in mouse models [30, 73]. However, the safety and scalability of these TLR-agonists may make their use in the clinic problematic. TLR 4 agonist adjuvants, in contrast, have been shown to be safe and effective in several clinical trials, and the TLR4 agonist adjuvant MPL is a component of the licensed HPV vaccine Cervarix^®^ (GlaxoSmithKline, Rixensart, Belgium).

In the current study, we have investigated the ability of a novel, fully synthetic lipid-A (SLA) TLR4 agonist to serve as an effective adjuvant when combined with the clinical stage antigen WN-80E [25, 26]. We find that SLA combined with either a stable oil-in-water emulsion (SE) or combined with Alum can induce a robust immune response to WN-80E, characterized by production of high level neutralizing antibodies. Furthermore, both of these formulations can affect antigen dose sparing and reduce the viral load in mice to undetectable levels following a single immunization compared to the same formulation without SLA. Investigation of cellular immune responses show that adjuvant formulations which reduce viral loads in mice also show increased levels of germinal center and, in some cases, plasmablast B-cells following immunization. Furthermore, inclusion of SLA increases the number of long lived antibody secreting cells in the bone marrow following a single immunization. These results highlight the versatility and utility of SLA as an adjuvant for WNV vaccines, and suggest a vaccine formulation whose components have a well documented safety profile for advancement into clinical testing.

## Materials and Methods

### Virus Stocks and Vaccines

Stocks of WNV (NY99 strain) were prepared from infected Vero cells (CCL-81, ATCC). Briefly, confluent cells were inoculated with WNV at a MOI of 0.1. Virus growth medium (MEM supplemented with 5% fetal bovine serum) was added to the flask after the virus was adsorbed onto drained monolayers for 60 minutes. Cells were examined daily following infection, and supernatant was harvested when cytopathic effect was evident throughout the culture. Decanted medium from the infected cells was clarified by centrifugation at 5000 x g for 10 min. Clarified supernatant was supplemented with additional FBS to a concentration of 15%. Virus was aliquoted and stored at -80C. Thawed stocks were titrated by plaque assay with titers of virus stocks typically 10^8^ pfu/ml.

The WN-80E protein utilized in these studies was provided by Hawaii Biotech, and has been previously described [26]. Briefly, the protein is a carboxy-truncated WNV E-protein which is produced in *Drosophila* S2 cells. Protein was provided in PBS, and stored at -80°C until use.

### Adjuvants and Immunogenicity Studies

All animal work described in this research was conducted under protocols approved prior to study initiation by the Infectious Disease Research Institute (IDRI) Institutional Animal Care and Use Committee (IACUC). The research conducted here was specifically approved by the IACUC.

SLA is a synthetic lipid-A derivative which is related to glucopyranosyl lipid A (GLA), which has been previously described [74]. For these studies, SLA was combined with Allhydrogel (SLA-Alum), combined with a stable oil-in-water emulsion (SLA-SE) containing squalene, or delivered as an aqueous formulation (SLA-AF).

For immunogenicity studies, 6–8 week old female C57Bl/6 mice were immunized via the intra-muscular route in a final volume of 100μl/immunization (50μL delivered to each leg) at 0 (prime) and 21 (boost) days. Seven days following each immunization serum, spleen and inguinal lymph nodes were collected for analysis. Twenty one days following each injection, additional serum and bone marrow were collected for analysis of WNV specific antibody titers and for ELISPOT analysis.

### Challenge Studies

Following immunization, 6–8 week old female C57Bl/6mice were challenged with 10^5^ plaque forming units (PFU) of WNV via intra-peritoneal injection of virus in 0.25 mL total volume. Following challenge, all animals were observed daily for signs of virus induced morbidity and mortality. 72 hours following challenge, peripheral blood was obtained from all animals via the retro-orbital sinus to determine serum virus titers. For survival studies, and per institutional IACUC guidelines, animals showing overt neurological symptoms including ataxia, decreased righting reflex, tremors, paralysis and others, or those which exhibit weight loss of more than 20% following challenge were euthanized by controlled CO_2_ inhalation.

### Plaque Assay

Serial 10 fold dilutions of serum were prepared in BA-1 medium (M-199 salts, 1.0% bovine serum albumin, 350 mg/L sodium bicarbonate, 100 units/mL penicillin, 100 mg/L streptomycin, and 1.0 mg/L amphotericin in 0.05 M Tris [hydroxymethyl aminomethane], pH 7.6) were prepared in 96 well plates (Corning). Diluted samples were added to 6-well (Corning) plates seeded 24 hours prior with 1 X 10^6^ Vero cells/well, and incubated for 60 minutes with shaking at 15 minute intervals to ensure even virus distribution. Wells were overlaid with a 0.5% agarose (Seakem) solution and incubated at 37°C for 48 hours to allow plaque formation. Following incubation, cells were stained with crystal violet to visualize and enumerate plaques.

### Plaque-Reduction Neutralization Test (PRNT)

Sera from immunized mice were inactivated by incubation at 56°C for 30 minutes. Inactivated sera was serially diluted 2-fold in BA-1 medium in a 96 well plate (Corning) beginning with a 1:5 dilution in a total volume of 100 μL. Following serum dilution, 100 μL of virus (200 pfu) was added to all serum samples. Virus:serum mixtures were incubated at 37°C for 60 minutes. Following incubation, virus in all samples was titrated using standard plaque assay techniques. Briefly, virus-serum mixtures were incubated with Vero cell monolayers (200 μL/well) at 37°C for 45 minutes with rocking to distribute the medium every 15 minutes. Wells were overlaid with 0.5% agarose and incubated for 2 days at 37°C in a CO_2_ incubator. Plaques were enumerated on day 3 following crystal violet staining. Negative (media only) and positive controls (immune serum) were included in each assay. Neutralizing antibody titers are given as the endpoint titer capable of reducing the number of plaques by 90% compared to a virus only control (PRNT_90_).

### Antibody responses

WN-80E-specific endpoint titers for IgG, IgG1 and IgG2c were determined seven days and twenty-one days post immunization. High binding polystyrene 384 well plates were coated with WN-80E (2 μg/ml) in 0.1 M bicarbonate coating buffer for 2.5 hours at room temperature. Plates were washed three times with 0.1% PBS—Tween 20 before and after a two hour blocking incubation with 0.05% PBS—Tween 20+1% BSA at room temperature. Mouse sera was serially diluted in 0.05% PBS—Tween 20+0.1% BSA using a Nanonscreen NSX-1536 and incubated overnight at 4°C and washed five times. Plates were incubated for 1 hour with anti-mouse IgG, IgG1 or IgG2c HRP conjugates (Southern Biotechnologies) with shaking. Following five washes, plates were developed by the addition of SureBlue tetramethylbenzidine substrate (Kirkegaard & Perry Laboratories) using a Nanoscreen robot. The enzymatic reaction was stopped with 1 N H_2_SO4 using a Multipette Sagian robot. Plates were read at 450–570 nm using the Synergy ELISA plate reader (Biotek) and Gen5 software.

### Antibody Secreting Cell ELISPOT Assay

WNV WN-80E specific antibody secreting cells present in the bone marrow were quantified using an ELISPOT assay. One day prior to assay initiation, Multiscreen ELISPOT plates (Millipore) were coated with 1ug of WN-80E/well, and incubated overnight. Blocked plates were washed three time with washing buffer (PBS + 0.5% Tween 20), blocked with collection medium for two hours, and washed 3 times. Bone marrow was collected 21 days post-immunization in RPMI medium supplemented with 10% fetal bovine serum (FBS), quantified using a Guava automated cell counter (Millipore) and resuspended to 1 X 10^6^ cells/mL. Cells were serially diluted 3-fold, added to plates, and incubated for 5 hours at 37°C. Secreted antibody was detected by addition of a 1:100 dilution of horse radish peroxidase (HRP) conjugated goat anti-mouse IgG antibody (Southern Biotech). Spots were visualized with an AEC Peroxidase substrate kit (Vector Labs) according to manufacturer’s instructions. Spots were quantitated on a CTL bioanalyzer.

### Intracellular cytokine staining

In order to quantify vaccine specific T-Cell responses, splenocytes were isolated from five mice per group following immunization. Red blood cells were lysed using Red Blood Cell Lysis Buffer (eBioscience) and resuspended in cRPMI 1640 (10% FBS, 1% Penicillin/Streptomycin; 0.1% 2-Mercaptoethanol). Cells were plated at 10^7^ cells/well in 96-well plates and were stimulated for 2 hours with media or WN-80E Antigen (10 μg/mL) at 37°C. At t = 2 hours, 1:50 GolgiPlug (BD Biosciences) was added and the cells were incubated for an additional 8 hours at 37°C. Cells were washed and surface stained with fluorochrome labeled antibodies (1:100 dilution in 1% BSA-PBS) to CD4 (clone RM4-5), CD8 (clone 53–6. 7), CD44 (clone IM7) and B220 (RA3-6B2) (BioLegend and eBioscience) in the presence of anti-CD16/32 (clone 93) for 15 minutes in the dark at room temperature. Cells were fixed and permeabilized with Cytofix/Cytoperm (BD Biosciences) for 30 minutes at room temperature in the dark. Cells were washed with Perm/Wash (BD Biosciences) and stained for 15 minutes with fluorochrome labeled antibodies to detect intracellular cytokines as follows: IFN-γ (clone XMG-1.2), IL-2 (JES6-5H4), TNF (MP6-XT22), IL-5 (clone: TRFK5) and IL-10 (clone: JES5-16E3) (BioLegend and eBioscience) Cells were washed, resuspended in 1% BSA-PBS and filtered using a 30-40um PP/PE 96 filter plate (Pall Corp). Up to 10^6^ events were collected on a four laser LSR Fortessa flow cytometer (BD Biosciences). Data were analyzed with FlowJo (Treestar).

### B cell quantification

Seven days following immunization, inguinal lymph nodes were isolated from five animals per group. Cells were re-suspended in cRPMI 1640 (10% FBS, 1% Penicillin/Streptomycin; 1:1000 2-Mercaptoethanol) and plated at 10^7^ cells/well in 96-well plates. Cells were surface stained in staining buffer (1% FBS, 1:250 EDTA, PBS) with fluorochrome labeled antibodies (1:200) to CD138 (clone281-2), GL7 (clone GL7), CD95 (clone Jo2), IgM (clone II/41), B220 (CloneRA3-6B2), IgD (clone 11-26c.2a), CD38 (clone 90) and 1:100 CD16/32 (clone 93) for 15 minutes in the dark at 4°C. Non B cell lineage cells (designated lineage-) were identified and excluded from analysis by staining (1:200) and gating for Ly6G (clone 1A8), CD11b (clone M1/70), CD11c (clone N418), F4/80 (clone BM8), Ter119 (clone TER-119) and Thy1.2 (clone 53–2.1) hi populations. Cells were fixed and permeabilized with Cytofix/Cytoperm (BD Biosciences) for 20 minutes at room temperature in the dark and washed with Perm/Wash (BD Biosciences). Cells were resuspended and filtered in staining buffer using a 30–40 μm PP/PE 96 filter plate (Pall Corp). Up to 10^6^ events were collected on a four laser LSR Fortessa flow cytometer (BD Biosciences). Data were analyzed with FlowJo (Treestar).

## Results

### SLA Stimulates Higher WNV Neutralizing Antibody Titers Following a Single Immunization in Mice

**Fig 1:**
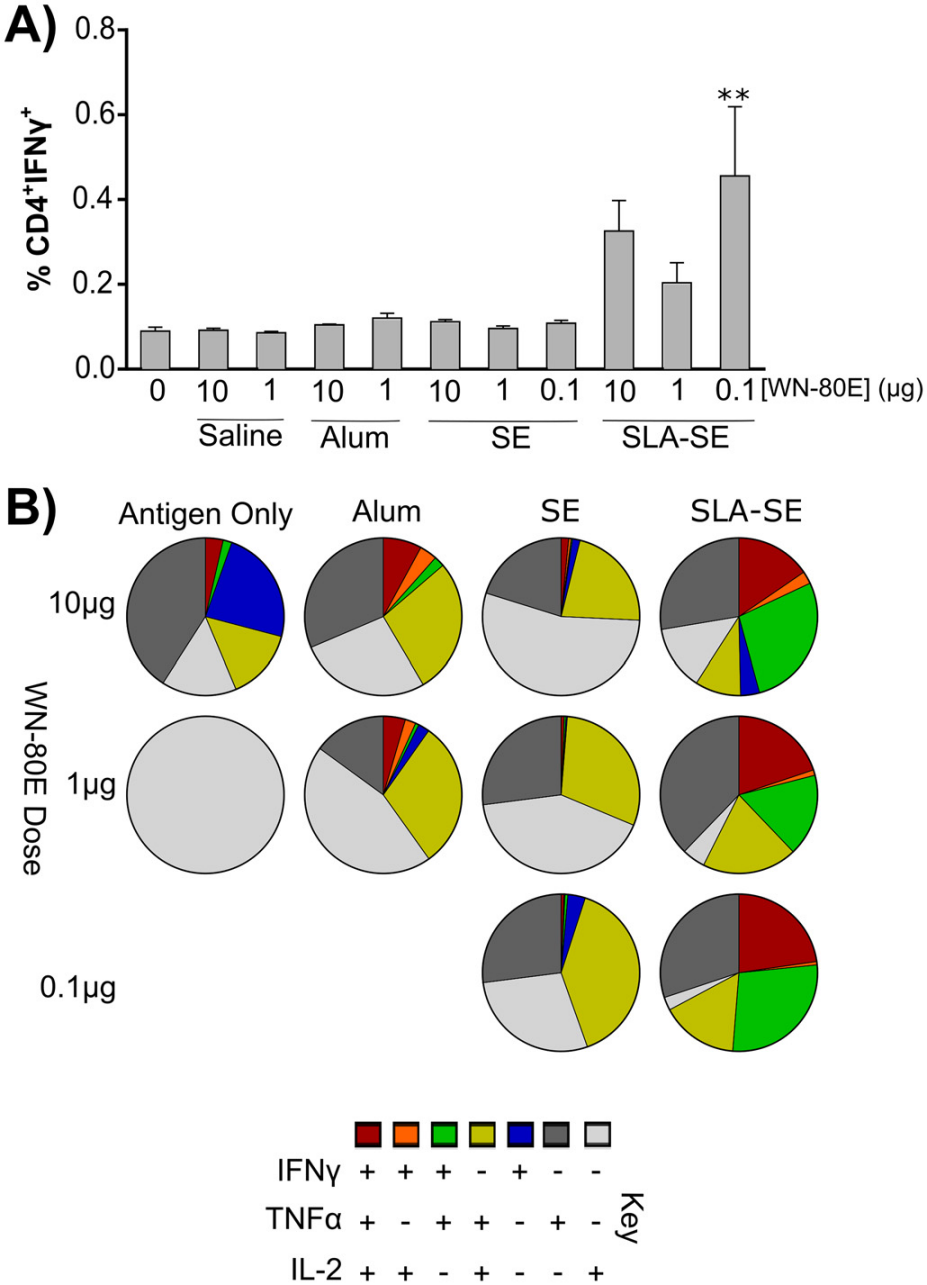
Induction of a Th1 CD4+ T-Cell Response in SLA-SE Immunized Animals. Seven days following a single immunization, isolated splenocytes (n = 5 mice/group) were phenotyped by ICS. IFNγ+ CD4 T-cells were induced following immunization with WN-80E in combination with SLA-SE. At decreased antigen doses (100 ng/mouse), inclusion of SLA-SE resulted in a significant increase in cytokine positive cells relative to antigen only controls (p<0.005). Additional cytokine profiling shows that many of the IFNγ cells in the SLA-SE group displayed a Th1 phenotype, and were positive for TNFα and/or IL-2 (B).

**Fig 2:**
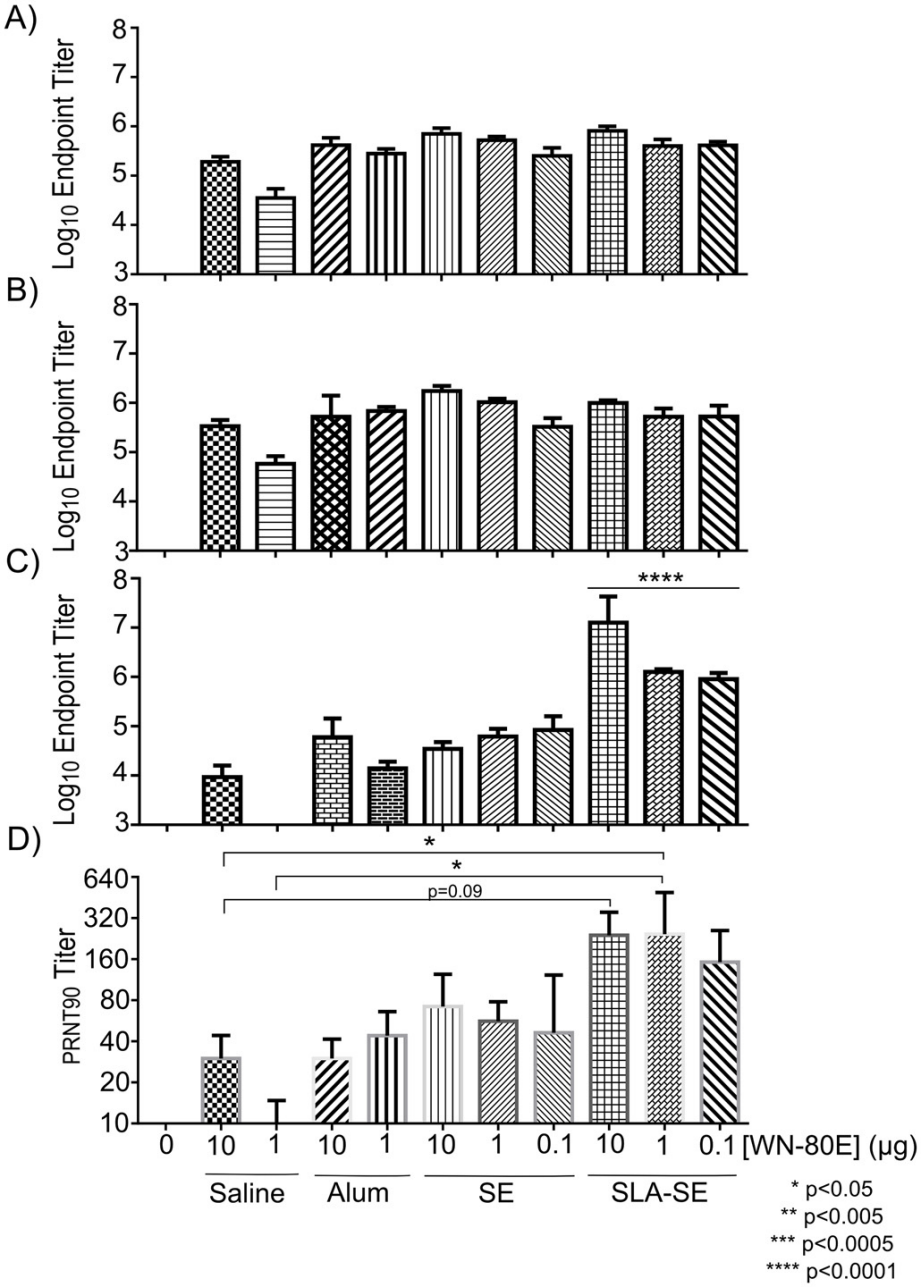
ELISA Titers Following A Single Immunization with WN-80E. Serum antibody titers were determined by ELISA 21 days following a single immunization with WN-80E in combination with adjuvants. Titers of Total IgG (A), IgG1 (B) and IgG2c (C) were determined for all mice (n = 5/group). One way ANOVA was used to evaluate significant differences in antibody levels and PRNT titers between groups. Similar levels of Total IgG and IgG1 were observed in all immunized animals. Significantly elevated levels of IgG2c were detected in mice immunized with SLA-SE compared to those immunized with 10 μg of antigen alone (p<0.0001). Neutralizing antibody titers were also determined by PRNT assay (D) to assess antibody function. There is a trend toward increased titer in SLA-SE immunized animals at all anitgen doses, and significant increases in PRNT titer are observed at the 1μg antigen dose.

In preliminary studies, we evaluated the ability of the TLR4 agonist adjuvant synthetic lipid A (SLA) formulated in a stable oil-in-water emulsion (SE) to enhance the immune response and enable antigen dose-sparing when combined with WN-80E. We utilized SE as a comparator due to widespread use of this class of adjuvants in commercial vaccines. In addition, we sought to compare these adjuvant formulations to WN-80E formulated with Alum, as this formulation has also been tested clinically. Following a single injection of WN-80E adjuvanted with alum, SE or SLA agonist combined with SE (SLA-SE), we examined both cellular and humoral WN-80E specific immune responses. Seven days following immunization, we observed an increase in the number of WN-80E specific IFNγ+ CD4+ T-cells in the spleen of SLA-SE immunized animals compared to those immunized with SE or alum (Fig 1). ICS analysis of T-cell populations demonstrated that many of these IFNγ^+^ T-cells secreted multiple cytokines, with a high percentage those from the SLA-SE immunized animals showing a canonical T_h_1 phenotype (IFNγ+/TNFα+/IL-2+) (Fig 1). The production of T_h_1 CD4+ T-cells at this timepoint was correlated with an increase in IgG2c antibodies in the serum 21 days post-immunization (Fig 2). In contrast, total IgG and IgG1 titers in serum at day 21 were similar among all adjuvanted groups (Fig 2). Examination of the neutralizing potential of the induced antibodies showed a correlation between the presence of IgG2c antibodies in the serum and increased neutralization potential; animals immunized with SLA-SE had the highest IgG2c titers and showed elevated PRNT titers compared to those immunized with alum or SE. Furthermore, high PRNT titers were induced even at relatively low antigen doses; the PRNT titers observed following immunization with 1 μg WN-80E + SLA-SE were significantly greater (P<0.05) than those observed following immunization with 1 μg of protein alone (Fig 1). Taken together, these results suggest that SLA-SE can increase the neutralizing antibody titer generated after a single injection with WN-80E, and that inclusion of the SLA agonist may allow up to 100 fold dose sparing of the antigen.

### SLA Can Enhance the Protective Efficacy of WN-80E In Multiple Formulations

**Fig 3:**
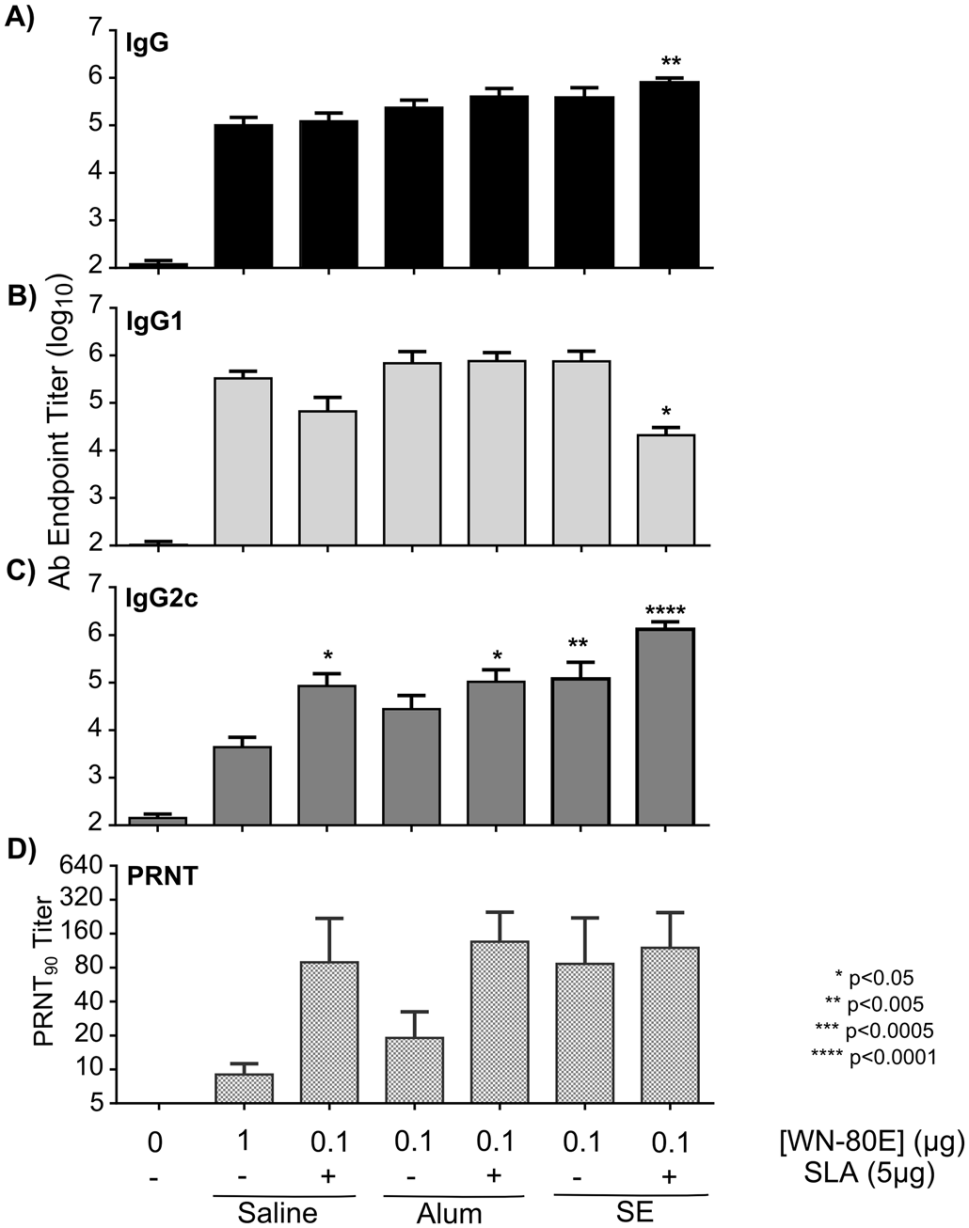
SLA Formulated with Alum or SE Increases Functional Antibody Titer Following A Single Immunization with WN-80E. Serum antibody titers were determined by ELISA 21 days following a single dose of WN-80E in combination with Alum or SE formulations with or without SLA. Anti-WN-80E titers of Total IgG (A), IgG1 (B) and IgG2c (C) were determined for all mice (n = 5/group). One way ANOVA was used to identify significant differences in antibody levels. Significantly elevated levels of IgG2c were detected in mice immunized with SLA containg adjvuants, as well as SE alone compared to those immunized with 1 μg of antigen alone. Mice immunized with SLA-SE showed the highest levels of IgG2c (p<0.0001). Mice receiving SLA-SE also had elevated levels of IgG2c relative to animals immunized with SE alone. Neutralizing antibody titers were also determined by PRNT (D) to assess antibody function. Elevated PRNT titers are observed in mice that received either SE alone, or SLA containing adjuvants in combination with WN-80E.

Given the increase in neutralizing antibodies induced by the combination of SLA and SE, we investigated whether or not addition of SLA could increase protective capacity when combined with the licensed adjuvant Alum. Mice were immunized once with reduced amounts (either 1 μg or 0.1 μg) of antigen in combination with stable emulsion or alum containing adjuvants via the intramuscular route. Five animals per group were euthanized 21 days following immunization to examine serum antibody responses to WN-80E, and the remaining animals (n = 10/group) were challenged via the intra-peritoneal route with 100 LD_50_ WNV (NY99 strain). Three days following challenge, serum was collected from all mice, and virus titers were determined by plaque assay. At day 21 post immunization all mice in adjuvanted groups induced similar levels of total serum IgG and IgG1 against WN-80E compared to antigen alone (Fig 3). As in the previous experiments, the inclusion of the SLA agonist adjuvant induced a significantly increased level of IgG2c when combined with both Alum and SE, as well as in an aqueous formulation (Fig 3). Those groups showing a significant increase in IgG2c titers also showed elevated PRNT titers at this timepoint (Fig 3). Consistent with previous work, immunization with SE alone also induced a neutralizing antibody response. These results are consistent with our previous findings, that SLA containing adjuvants show increased neutralizing potential, and that this is correlated with the induction of a Th1 antibody response characterized by increased levels of IgG2c.

**Fig 4:**
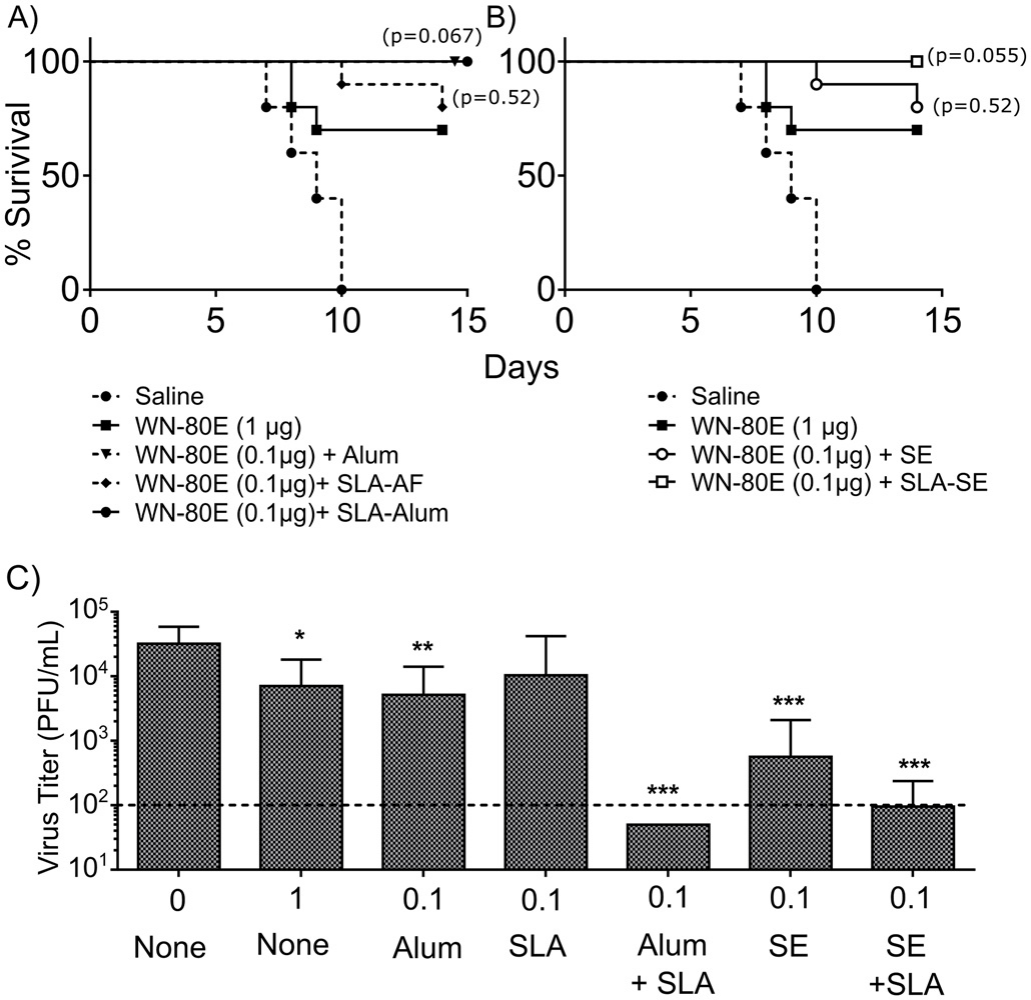
Immunization with SLA Containing Adjuvants in Combination with WN-80E Enhances Survival and Reduces Viral Titer to Undetectable Levels. Following a single immunization of WN-80E in combination with the indicated adjuvants, mice (n = 10/group) were challenged with 100 LD_50_ of WNV via the intraperitoneal route. Surivial of mice was monitored over 14 days following challenge (A,B). Survival curves were compared using a Mantel-Cox test, with p-values compared to immunization with WN-80E shown. Three days post-challenge, serum was collected from all animals in order to assess virus titers. Animals immunized with SLA-Alumhad undetectable titers in all animals (P<0.0005). Those imminzed with SE or SLA-SE had minimal titers while those immunized with Alum, SLA-AF or no adjuvant showed only slightly reduced titers compared to unimmunized controls (P<0.05).

In addition to serologic evaluation, we investigated the ability of SLA containing adjuvants to protect animals from lethal WNV challenge following a single immunization. Mice were immunized once with WN-80E with or without adjuvant, and challenged 21 days post-immunization. Following challenge, all control mice succumbed to infection by day 10. Consistent with previous data utilizing WN-80E, mice immunized with 1μg antigen alone showed a 70% survival rate, while 80% of animals immunized with WN-80E combined with either SE emulsion alone or an aqueous formulation of SLA (SLA-AF) survived. All animals immunized with Alum, SLA-Alum or SLA-SE adjuvants survived challenge (Fig 4, Table 1). Comparison of survival curves show that survival in animals immunized with 100ng of WN-80E combined with adjuvant is equivalent to that observed with 1 μg of antigen alone.

**Table 1:** Survival and Viral Titers Following WNV Challenge.

Antigen	Dose	Adjuvant	Survival	Animals With Detectable	Day 3 Virus Titer	
	(μg)			Virus Titer (%)^a^	Average	(Range)
None	0	None	0/10	100	3.2 X 10^4^	(3 X 10^3^–5 X 10^4^)
WN-80E	1	None	7/10	100	7.1 X 10^3^	(2 X 10^2^–5 X 10^4^)
WN-80E	0.1	Alum	10/10	70	5.1 X 10^3^	(<100–2.9 X 10^4^)
WN-80E	0.1	SLA	8/10	40	3.7 X 10^2^	(<100–2.9 X 10^4^)
WN-80E	0.1	Alum + SLA	10/10	0	<1.0 X 10^2^^b^	(<100)
WN-80E	0.1	SE	8/10	30	5.7 X 10^2^	(<100–4.9 X 10^4^)
WN-80E	0.1	SE + SLA	10/10	10	9.5 X 10^1^	(<100–5 X 10^2^)

^a^ Virus titer was determined on d3 post-challenge

^b^ Virus titers in this group were below the limit of detection.

In addition to survival, we have examined the viral titers 3 days following challenge, and find that adjuvants were variably effective in reducing viral load (Fig 4). Animals immunized with 0.1 μg WN-80E and Alum or SE alone showed detectable titers in 70% and 30% of animals. Addition of SLA to SE reduced the number of animals with detectable titer to 10%, while addition of SLA to Alum resulted in no detectable virus titer in any animal at this time point. Collectively, these results demonstrate that addition of the TLR agonist SLA in formulations can enhance the protection of WN-80E antigen in mice by reducing serum virus titers to minimal or undetectable levels at a low antigen dose (0.1 μg) after only a single immunization.

### SLA Induces an Increase in IgG^+^ Antibody Secreting Cells In The Bone Marrow Following A Single Immunization

**Fig 5:**
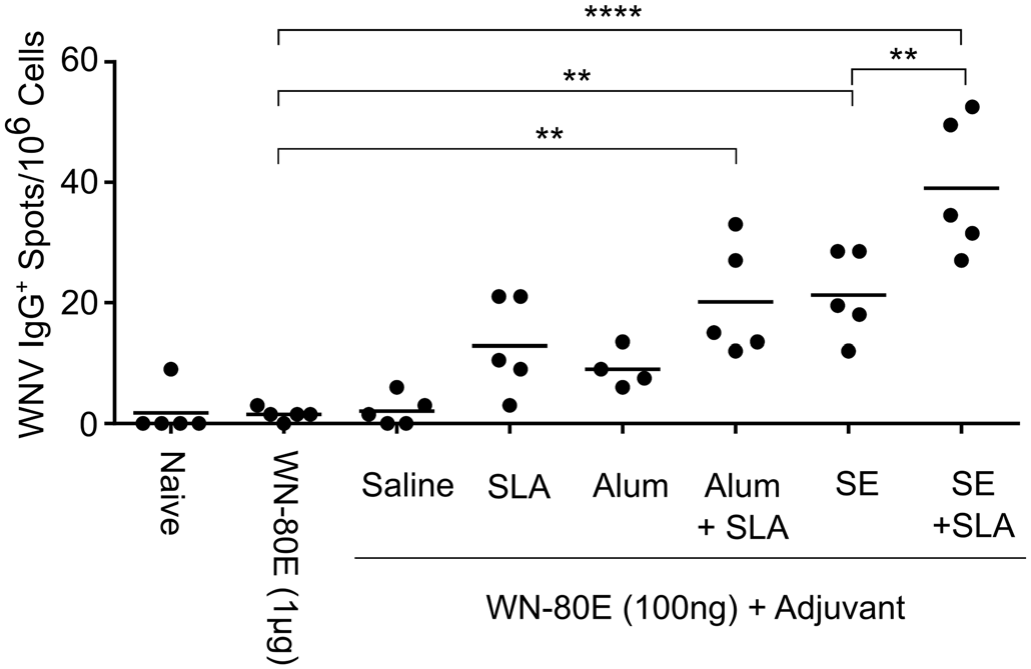
Immunization with SLA Containing Adjuvants in Combination with WN-80E Induces Antibody Secreting Cells in Bone Marrow. In an independent experiment, we have examined the ability of WN-80E combined with SLA containing adjuvants to induce long lived antibody secreting cells (ASC) in bone marrow. Mice were immunized and bone marrow extracted after 21 days (n = 5/group). The number of antibody secreting cells was assessed by ELISPOT assay on plates coated with WN-80E. Differences between groups were compared by one way ANOVA. Adjuvant formulations containing SLA (SLA-Alum, SLA-SE) as well as emulsion alone induced significantly greater numbers of IgG+ ASC compared with antigen alone. Animals immunized with Alum or unformulated SLA also showed modest increases in numbers of ASC, but differences were not significant relative to antigen only.

In addition to stimulating a robust acute phase protective response, effective vaccines must also stimulate durable long term immunity. To investigate the ability of SLA to enhance durable responses to WN-80E, we have investigated the presence of WNV E-protein specific IgG antibody secreting cells (ASC) in the bone marrow following a single immunization (Fig 5). These long-lived cells are a correlate of durable immunity due to their ability to continually produce and secrete antibodies. To quantify WNV specific ASC, bone marrow was collected 21 days following immunization, and ASC quantified using an ELISPOT assay. SLA containing adjuvants, in addition to SE, induced increased numbers of IgG+ ASC relative to antigen alone. Addition of SLA to SE resulted in further significant increases in ASC numbers. Overall, adjuvants which were capable of reducing serum virus titer also stimulated production of ASC, demonstrating their potential to generate long-term durable immunity.

### SLA Induces an Increase in Germinal Center B-Cells and Plasmablasts Following Immunization

**Fig 6:**
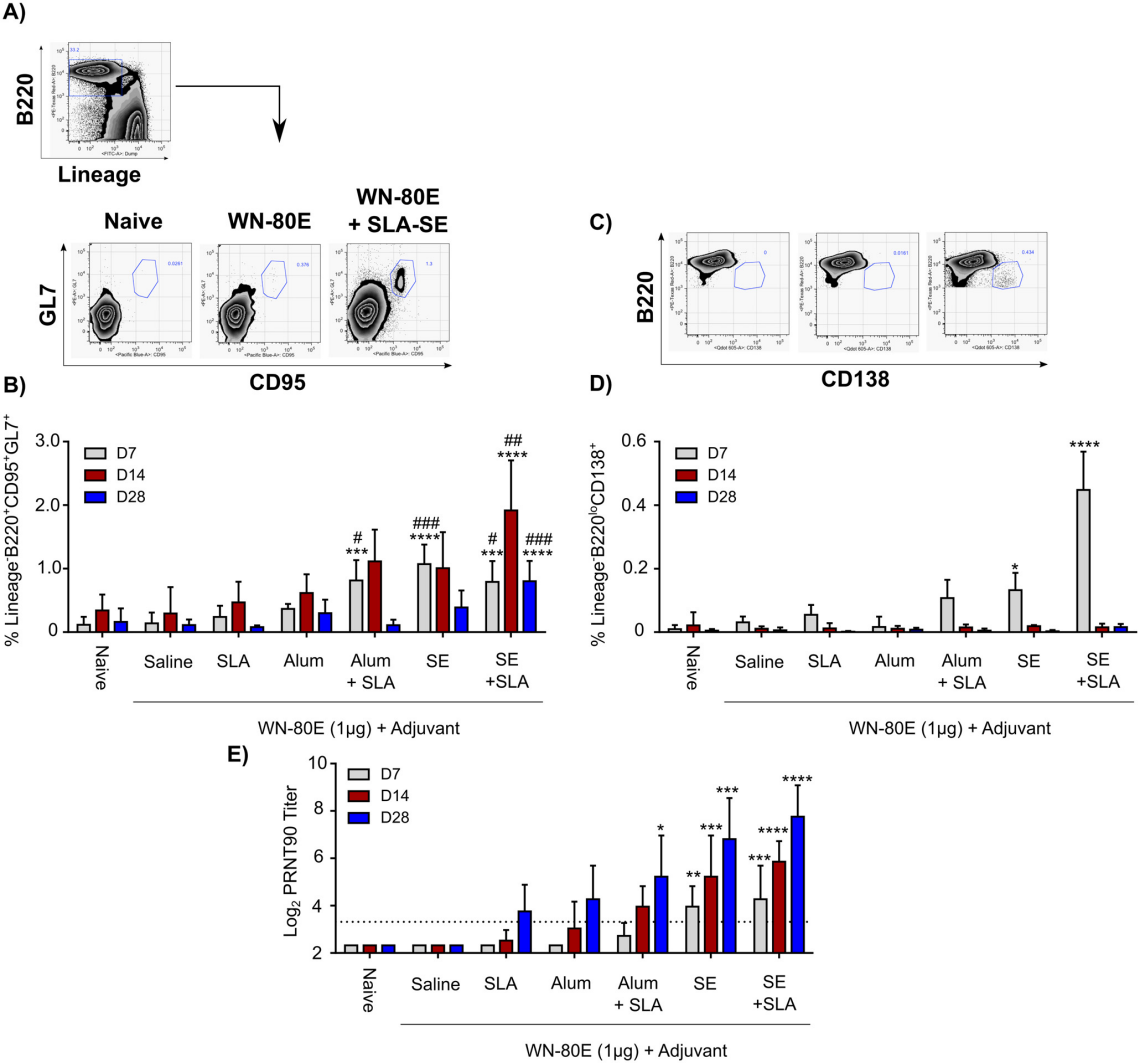
Vaccine Formulations which reduce WNV titer post-challenge induce germinal center B-cells and plasmablasts following immunization. Animals were immunized once with WN-80E (1ug) in combination with Alum or SE with or without SLA. Over a timecourse following this immunization, inguinal lymph nodes were removed and stained for B cell markers by ICS. Gating strategies for germinal center B-cells (A) and plasmablasts (C) are shown. Immunization with SLA-Alum, SE, or SLA-SE resulted in a sustained increase in the number of germinal center B-cells (CD95^+^GL7^+^) (B). Increased numbers of cells were observed relative to both WN-80E alone (***P<0.0005, **** p<0.0001) and relative to WN-80E combined with Alum (# p<0.05, ### p<0.0005) at the same timepoint. Numbers of germinal center cells peaked at D14, and declined by D28 post-immunization. Elevated numbers of plasmablast cells (CD138+B220lo) were also observed at day 7 post-immunization, but declined thereafter (D). Increases in germinal center cell numbers resulted in significant increases in PRNT titers as early as 7 days post immunization (E). Differences between groups were determined by one-way ANOVA using Dunnett’s Multiple comparison test.

The previous experiments demonstrate the utility of the TLR4 agonist SLA as an adjuvant for a single-shot WNV vaccine in multiple formulations, and show that the SLA-Alum and SLA-SE formulations provide robust immunity insofar as minimal virus could be detected on day 3 post-challenge in the majority of challenged animals. In a second independent study, we further investigated the cellular correlates for reduction of day 3 post-challenge serum virus titers observed in our study (Fig 6 and S1 Fig). We observe a statistically significant increase in GC B-cells following immunization with formulations which show the lowest serum virus titers following challenge; SLA-Alum, SE, and SLA-SE (Fig 6). Increased germinal centers could be observed as early as 7 days post-immunization, reached a peak 14 days post-immunization, and declined by day 28. SLA-Alum, SE and SLA-SE all showed statistically significant increases over both WN-80E alone and WN-80E + Alum at 7 days post immunization. Of the formulations tested, SLA-SE induced the greatest numbers of GC B-cells, which were statistically increased compared with WN-80E immunization at all timepoints. This formulation also induced a significant number of plasmablast (CD138+B220lo) cells at 7 days, while more modest increases were observed with SE and SLA-Alum formulations. Plasmablasts were present at day 7, but were absent at days 14 and 28.

Increases in GC B cells correlated with statistically significant increases in PRNT titers over the same timecourse. Formulations which showed a statistically significant increase in GC cells at early timepoints showed significantly increased PRNT by day 28 post-immunization. Emulsion based formulations, which showed increased GC B-cells and, in the case of SLA-SE, increases in plasmablast B-cells, showed a more rapid elevation of serum neutralizing titers, with statistically increased PRNT titers 7 days post-immunization.

## Discussion

There are a number of WNV vaccines in pre-clinical or clinical stages of development, yet to date, none are available for human use. Live attenuated WNV vaccines based on the 17D strain of Yellow Fever virus have advanced the furthest in clinical trials; the vaccine has shown positive safety and immunogenicity profiles in Phase I and Phase II trials [62, 64, 75]. However, as with all live attenuated vaccines, the ability of the vaccine vector to replicate in immunized subjects and potentially to cause disease during the viremic period remains a concern [76]. Furthermore, live attenuated vaccines such as Yellow Fever pose a more significant risk to elderly and immunocompromised individuals, who are at greater risk for severe complications from WNV infection [77–79]. In order to circumvent some of these safety concerns, a number of subunit vaccines based on the E protein have been developed. Of these, the WN-80E protein is the most clinically advanced; WN-80E was shown in a phase I clinical study to be safe and immunogenic after 3 doses of 5 μg, 15 μg or 50 μg of protein adjuvanted with Alhydrogel (Clinical Trial#: NCT00707642). While these results are promising, the overall level of virus neutralizing antibody induced by this vaccine was low relative to live attenuated vaccines. The primary goal of this study was to identify an adjuvant which may provide both dose and dosage sparing functions, ultimately enabling durable protection following a single dose of WN-80E antigen at levels similar to those observed with live attenuated vaccines.

In pre-clinical development studies with WN-80E, 1 μg of protein was shown to be immunogenic in mice following two injections with the saponin based ISCOMATRIX^®^ adjuvant [26]. In this study, we have demonstrated induction of PRNT titers in mice following a single injection of 0.1 μg of WN-80E in combination with SLA-SE. The level of neutralizing antibody following immunization, which serves as a correlate of protection for several other Flavivirus vaccines, was dependent on the presence of SLA, and was correlated with an increase in serum IgG2c titers. The induction of IgG2c antibodies is in turn dependent on induction of a Th1 CD4+ T-cell response by SLA, providing a mechanism for SLA mediated enhancement of protection that is consistent with studies investigating other vaccines [80, 81]. In an additional arm of this study, we have boosted the response in all groups with an additional injection, and find that PRNT titers as well as IgG2c levels are increased in all adjuvanted groups (S2 Fig), including those which do not contain SLA.

The enhancement of neutralizing antibody responses by SLA-SE prompted us to examine the ability of SLA to enhance antigen specific responses in additional formulations. While emulsion based adjuvants (e.g. MF95, Novartis) are widely demonstrated to be effective and are in use clinically in Europe, approval in the US and other countries has been problematic to date. In order to initiate development of a vaccine formulation that may be advanced into clinical trials, we focused on SLA-Alum for two reasons. First, WN-80E has already shown promise in clinical trials in combination with Alum. Second, the SLA-Alum formulation utilized in this study is similar to AS04 (GlaxoSmithKline), which combines the TLR-4 agonist monophosphoryl Lipid-A (MPL) and Alum, and which is licensed for use as a component of the HPV vaccine Cervarix^®^. The primary difference between SLA-Alum and AS04 is the use of a fully synthetic, rationally designed TLR4 agonist (SLA) which has improved potency compared to a purified biological product (MPL) which is a mixture of compounds, only some of which show TLR-4 agonism in humans [72]. As with SLA-SE, we find that SLA-Alum is capable of increasing the neutralizing antibody response following a single immunization with WN-80E, with the magnitude of the neutralizing response similar between SLA-SE and SLA-Alum at a low antigen dose.

As expected from previous studies [26], immunization with WN-80E alone or combined with Alum resulted in significant increases in survival of animals, a finding consistent with the relatively low lethality of WNV in murine models and previously published work describing WN-80E. While the mechanism of this protection is unclear, lethality following WNV infection is associated with infection of the central nervous system (CNS), which may not occur in all infected animals following peripheral WNV challenge [8, 82]. Previous studies conducted in a hamster challenge model with WN-80E have shown complete protection of all immunized animals, despite very low PRNT titers [83]. Our findings are similar insofar as animals with low neutralizing antibody titers have survived challenge. While there was a trend toward increased survival of animals immunized with SLA-containing vaccines in our challenge studies, these do not reach statistical significance.

In contrast to survival data, there was a marked difference in the ability of adjuvant formulations to reduce serum viral titer at early times post challenge; in this case, the inclusion of SLA, particularly to Alum, resulted in dramatic decreases in viral serum virus titer. At low antigen doses (100ng), immunization with alum resulted in an average titer decrease of less than 10-fold relative to naïve controls, with 70% of animals showing measurable virus titers. Addition of SLA to Alum resulted in undetectable virus in 100% of animals, which represents a decrease in titer of approximately 1000-fold relative to uninfected controls. All of the emulsion based formulations tested (SE, SLA-SE) were capable of reducing virus titer, a finding which is consistent with many studies demonstrating the ability of emulsion based adjuvants to enhance protective antibody responses.

In addition to acute phase antibody responses, we have examined the ability of adjuvant formulations to induce long term immunity (Fig 5). In this case, inclusion of SLA increased the number of WNV-specific IgG+ ASC regardless of formulation. This finding is consistent with the ability of SLA to improve durability of immune responses to WN-80E, although a long term study would be required to assess protection from WNV challenge at timepoints distant from immunization.

Examination of cellular responses following a single immunization demonstrates that those adjuvant formulations capable of reducing WNV titer had increased germinal center B-cells in draining lymph nodes as early as 7 days post immunization (Fig 6). These cells reached a peak at 14 days post immunization and declined thereafter. Importantly, those formulations showing the lowest serum virus titer (SLA-Alum, SE, SLA-SE) also showed significantly increased titers relative to WN-80E + Alum. The pattern of serum neutralizing antibodies observed in in this study correlated with GC induction; those formulations which generated an increased germinal center response had significantly increased PRNT titers by d28 post-challenge. In previous studies, the inclusion of GLA, a TLR-4 agonist adjuvant similar to SLA, was shown to increase the diversity of antibody variable regions following immunization with a malaria antigen [84], suggesting a TLR-dependent maturation of the antibody response which correlates with increased neutralization potential. Future studies are planned with WN-80E which directly address the antibody sequence diversity induced by protective adjuvant formulations, and these will assess the contribution of both germinal center and plasmablast B-cells to the neutralizing antibodies induced by vaccination. In addition, characterization of the binding sites of novel antibodies induced by SLA-SE may allow identification of new important antibody binding sites in the WNV E protein. Previous studies have mapped neutralizing antibodies to epitopes in DII and DIII in both WNV as well as other flaviviral E proteins in mice (reviewed in [15]). However, more recent studies suggest that DIII antibodies may not play a critical role in neutralization in humans infected with other flaviviruses [85].

Another promising aspect of these results is the possibility of broadened protection against diverse flaviviruses induced by SLA-Alum or SLA-SE. Many studies have previously investigated cross-protection capability between flavivirus E-proteins, and have found that E-proteins from one virus can protect against other viruses in the genus [86, 87]. This cross protection is attributed to structural similarities between the E-proteins of members of a flavivirus serogroup. In previous studies with other viruses such as highly pathogenic avian influenza (HPAI), TLR-4 agonist adjuvants have been shown to increase protection not only to homologous virus, but also to antigenically distinct heterologous viruses [81, 88]. These findings, in combination with those presented here suggest the possibility that SLA containing adjuvants represent a tool to enhance protection against drifted flaviviral strains, such as the lineage 2 WNV viruses which are currently emerging in Europe [89–91]. SLA based formulations may also be useful to enhance the protection across the four dengue virus (DENV) serotypes, where protection against the multiple serotypes is critical for an effective vaccine.

In summary, we have utilized a clinical stage recombinant WNV antigen, WN-80E, to identify SLA adjuvant formulations capable of generating robust immune responses. The results demonstrate that robust responses can be generated after a single dose and these responses protect against virus challenge in the mouse model of West Nile Viral disease. Furthermore, we demonstrate that SLA-Alum induces enhanced protection in mice when compared to Alum alone, as no virus was detected by the plaque method in any of the mice in the SLA-Alum group. Future work to optimize this formulation by investigating additional doses of SLA and routes of immunization will provide a foundation for advancement of this vaccine into additional models and future clinical studies. Ultimately, the use of SLA as an adjuvant may provide a more effective vaccine for this emerging public health threat and help to reduce the severity and size of future WNV outbreaks.

## Supporting Information

S1 Fig Protective Adjvuants Induce a Th1 CD4+ T-Cell response following a single immunization. Mice (n = 5/group) were immunized with WN-80E (1 μg/dose) in combination with the indicated adjuvants. 7 days following a single immunization, splenocytes were isolated and phenotyped by ICS. SLA-SE which is shown to reduce serum virus titer in challenge studies induced an increased number of CD4+ T-cells with a Th1 phenotype (A), and many of these were also positive for other Th1 cytokines including TNFa and IL-2 (B).
(TIF)

S2 Fig Induction of WN-80E Specific Antibodies in Serum Following Two Injections With WN-80E. Serum antibody titers were determined by ELISA 21 days following a boost immunization with WN-80E in combination with adjuvants. Titers of Total IgG (A), IgG1 (B) and IgG2c (C) were determined for all mice (n = 5/group). Similar levels of Total IgG and IgG1 were observed in all immunized animals. Significantly elevated levels of IgG2c were detected in mice immunized with all adjuvants compared to those immunized with 10 μg of antigen alone. Unlike results obtained following a single injection, IgG2c levels were elevated in all animals receiveing adjuvant relative to those receiving antigen only. Neutralizing antibody titers, determined by PRNT assay (D), were also elevated in all animals receiving adjuvant.
(TIF)
