# Supplementary figures and images for: A Novel Synthetic TLR-4 Agonist Adjuvant Increases the Protective Response to a Clinical-Stage West Nile Virus Vaccine Antigen in Multiple Formulations
Source: PLoS One. 2016 Feb 22;11(2):e0149610. doi: 10.1371/journal.pone.0149610 (PMC4762984; doi:10.1371/journal.pone.0149610)

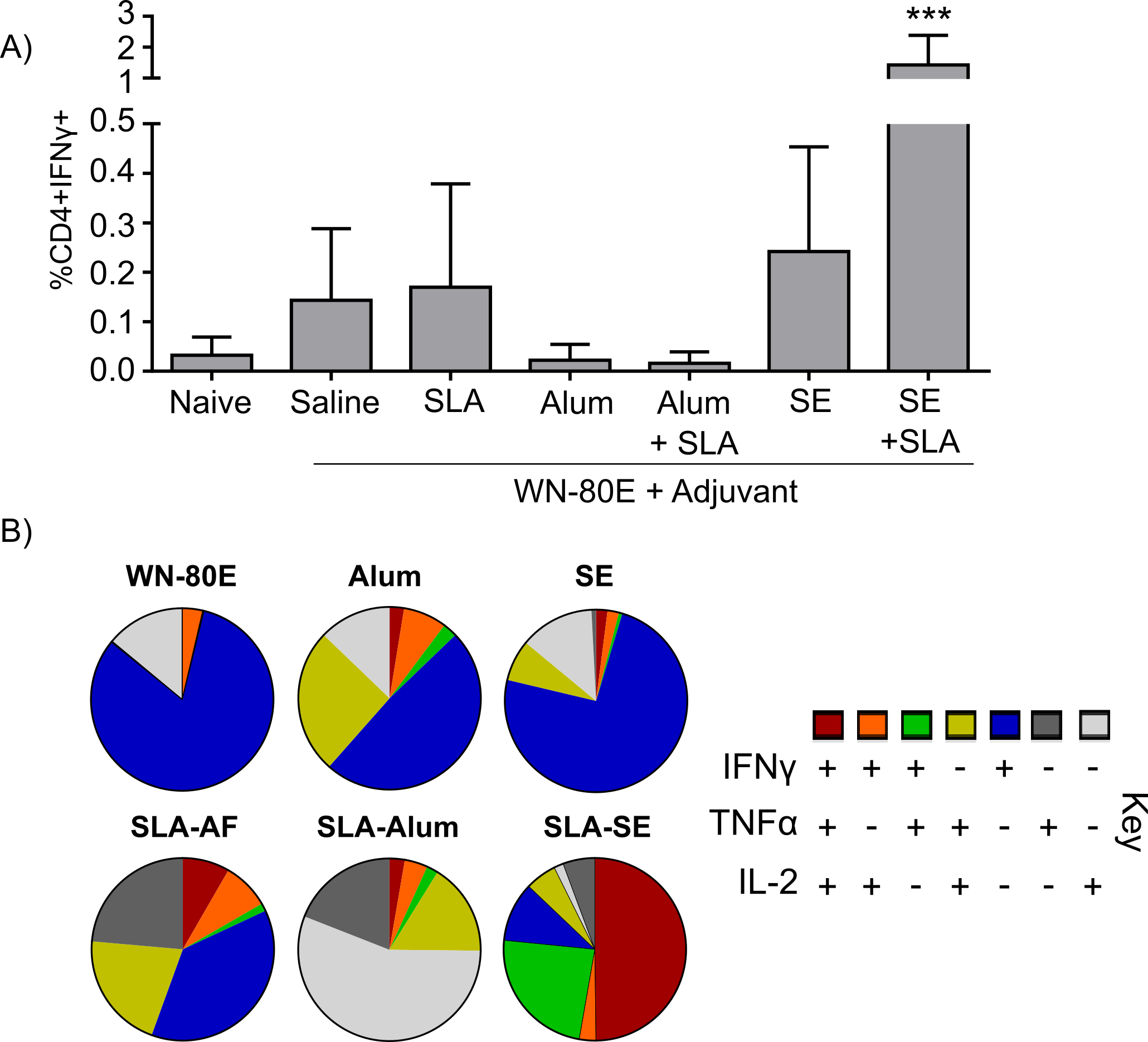

Supplement: S1 Fig — Mice (n = 5/group) were immunized with WN-80E (1 μg/dose) in combination with the indicated adjuvants. 7 days following a single immunization, splenocytes were isolated and phenotyped by ICS. SLA-SE which is shown to reduce serum virus titer in challenge studies induced an increased number of CD4+ T-cells with a Th1 phenotype (A), and many of these were also positive for other Th1 cytokines including TNFa and IL-2 (B). (TIF) [file pone.0149610.s001.tif]

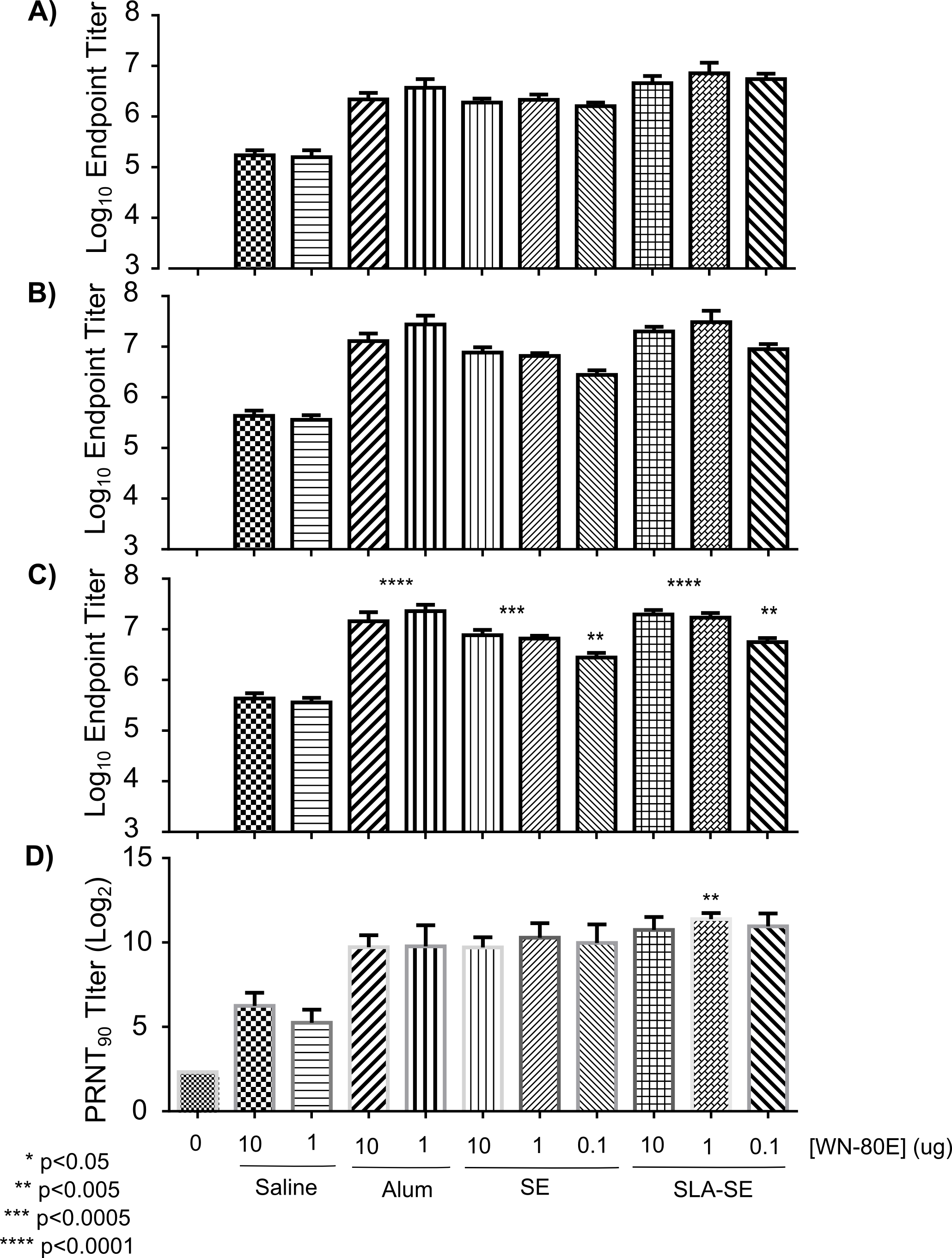

Supplement: S2 Fig — Serum antibody titers were determined by ELISA 21 days following a boost immunization with WN-80E in combination with adjuvants. Titers of Total IgG (A), IgG1 (B) and IgG2c (C) were determined for all mice (n = 5/group). Similar levels of Total IgG and IgG1 were observed in all immunized animals. Significantly elevated levels of IgG2c were detected in mice immunized with all adjuvants compared to those immunized with 10 μg of antigen alone. Unlike results obtained following a single injection, IgG2c levels were elevated in all animals receiveing adjuvant relative to those receiving antigen only. Neutralizing antibody titers, determined by PRNT assay (D), were also elevated in all animals receiving adjuvant. (TIF) [file pone.0149610.s002.tif]
